# Fauna Europaea: Annelida - Terrestrial Oligochaeta (Enchytraeidae and Megadrili), Aphanoneura and Polychaeta

**DOI:** 10.3897/BDJ.3.e5737

**Published:** 2015-09-11

**Authors:** Emilia Rota, Yde de Jong

**Affiliations:** ‡ University of Siena, Siena, Italy; § University of Amsterdam - Faculty of Science, Amsterdam, Netherlands; | Museum für Naturkunde, Berlin, Germany

**Keywords:** Biodiversity Informatics, Fauna Europaea, Taxonomic indexing, Zoology, Biodiversity, Taxonomy, Annelida, Oligochaeta, terrestrial, Megadrili, Enchytraeidae, Propappidae, Aphanoneura, non-marine Polychaeta

## Abstract

*Fauna Europaea* provides a public web-service with an index of scientific names (including important synonyms) of all living European land and freshwater animals, their geographical distribution at country level (up to the Urals, excluding the Caucasus region), and some additional information. The *Fauna Europaea* project covers about 230,000 taxonomic names, including 130,000 accepted species and 14,000 accepted subspecies, which is much more than the originally projected number of 100,000 species. This represents a huge effort by more than 400 contributing specialists throughout Europe and is a unique (standard) reference suitable for many users in science, government, industry, nature conservation and education.

This paper provides updated information on the taxonomic composition and distribution of the Annelida - terrestrial Oligochaeta (Megadrili and Enchytraeidae), Aphanoneura and Polychaeta, recorded in Europe. Data on 18 families, 11 autochthonous and 7 allochthonous, represented in our continent by a total of 800 species, are reviewed, beginning from their distinctness, phylogenetic status, diversity and global distribution, and following with major recent developments in taxonomic and faunistic research in Europe. A rich list of relevant references is appended. The Fauna Europaea Annelida - terrestrial Oligochaeta data-set, as completed in 2004, will be updated accordingly.

## Introduction

The European Commission published the European Community Biodiversity Strategy, providing a framework for development of Community policies and instruments in order to comply with the Convention on Biological Diversity. This Strategy recognises the current incomplete state of knowledge at all levels concerning biodiversity, which is a constraint on the successful implementation of the Convention. Fauna Europaea contributes to this Strategy by supporting one of the main themes: to identify and catalogue the components of European biodiversity into a database in order to serve as a basic tool for science and conservation policies.

With regard to biodiversity in Europe, both science and policies depend on a knowledge of its components. The assessment of biodiversity, monitoring changes, sustainable exploitation of biodiversity, and much legislative work depend upon a validated overview of taxonomic biodiversity. Towards this end Fauna Europaea plays a major role, providing a web-based information infrastructure with an index of scientific names (including important synonyms) of all living European land and freshwater animals, their geographical distribution at country level and some additional useful information. In this sense, the Fauna Europaea database provides a unique reference for many user-groups such as scientists, governments, industries, conservation communities and educational programs.

Fauna Europaea started in 2000 as an EC-FP5 four-years project, delivering its first release in 2004. After Fifteen years of steady progress, in order to efficiently disseminate the Fauna Europaea results and to increase the acknowledgement of the Fauna Europaea contributors, novel e-Publishing tools have been applied to prepare data-papers of all major taxonomic groups. For this purpose a special Biodiversity Data Journal Series has been compiled, called Contributions on Fauna Europaea. This work was initiated during the ViBRANT project and is further supported by the recently started EU BON project. This paper holds the first publication of the Fauna Europaea Annelida-Terrestrial Oligochaeta (Enchytraeidae and Megadrili), Aphanoneura and Polychaeta data sector as a BDJ data paper.

Within the EU BON project also further steps will be made to implement *Fauna Europaea* as a basic tool and standard reference for biodiversity research and to evaluate taxonomic expertise capacity in Europe. The *Fauna Europaea* data-papers will contribute to a quality assessement on biodiversity data by providing estimates on gaps in taxonomic information and knowledge.

## General description

### Purpose

The Fauna Europaea is a database of the scientific names and distribution of all living, currently known multicellular European land and fresh-water animal species assembled by a large network of experts, using advanced electronic tools for data collations and validation routines. An extended description of the Fauna Europaea project backgrounds can be found in Jong et al. 2014, a summary is given in the sections below.

The Annelida-terrestrial Oligochaeta (Megadrili and Enchytraeidae), Aphanoneura and Polychaeta is one of the 58 Fauna Europaea major taxonomic groups. In the first release of the FaEu database (2004) it covered 735 species.

### Additional information

**Introduction to Annelida-terrestrial Oligochaeta, Aphanoneura and Polychaeta.**

**Part 1: Aphanoneura and Polychaeta**

**Class** APHANONEURA

Two families whose sister-group relationship is supported by morphological and molecular data (Purschke and Hessling 2002, Marotta et al. 2003, Struck and Purschke 2005). Similar to clitellates in lacking parapodia, being hermaphroditic, and laying cocoons secreted by a specialized area of the body wall, features that for long time have suggested their inclusion in the Oligochaeta, either as the most primitive group or as a secondarily simplified, derived branch. However, the possession of nuchal organs (Fauchald and Rouse 1997, Hessling and Purschke 2000), the construction of the central nervous system (Purschke and Hessling 2002) and pharynx (Bunke 1967), and the ultrastructure of spermatozoa (Bunke 1985, Bunke 1986, Marotta et al. 2003) exclude any close affinities with clitellates. Molecular studies (Rota et al. 2001, Struck and Purschke 2005, Rousset et al. 2007) indeed indicate that the aphanoneurans are not closer to clitellates than any other annelid grouping.

**Family Aeolosomatidae**

Cosmopolitan family of minute worms living interstitially or epibenthically mostly in freshwater habitats. Some also occur in damp soils and forest litter; others (*Hystricosoma*, *Aeolosoma* spp.) live epizoically on freshwater crayfish; one species is marine. Represented in Europe by 3 genera (*Aeolosoma*, *Hystricosoma*, *Rheomorpha*) and about 25 species. Most *Aeolosoma* species are recognizable by possessing scattered epithelial “oil glands” that are red, green or yellow *in vivo*, dorsal and ventral bundles of capillary chaetae, and a ciliated prostomium used for locomotion and suction-feeding. *Hystricosoma* moves by muscular contractions of the body wall, has only sigmoid chaetae and those of each dorsal bundle emerge as two parallel rows running in opposite directions; the orange-red oil glands are concentrated around the dorsal chaetal bundles. *Rheomorpha* has pale greenish oil glands and bears adhesive epidermal papillae in place of the chaetae and at the tip of the bilobed pygidium. Testes develop in both anterior and posterior segments; mature sperm are released through the nephridia and transferred into 2-5 pairs of simple ventral epidermal invaginations of the partner. Eggs are laid inside cocoons secreted by a glandularized epithelium that extends for one segment laterally and ventrally beneath the single mature ovary; the unpaired female pore opens in the middle of this pseudo-clitellum. Sexual reproduction, however, is rarely observed, reproduction most often implies paratomic formation of chains of 2-8 zooids.

**Family Potamodrilidae**

Monotypic family, with one species in Europe (*Potamodrilus fluviatilis*) and one undescribed congener (*Potamodrilus* sp.) in North America (Strayer 2001; Roanoke River, Virginia, acc. to Smithsonian Museum Collection). The minute body has no epidermal oil glands and comprises only six chaetigerous segments, each bearing paired dorsolateral and ventrolateral couples of hair chaetae (one long and one short). Locomotion is due to a three-lobed ventral ciliary field on prostomium, but ventral longitudinal body wall muscles allow rapid curling of the body. Pygidium narrow, funnel-shaped, adhesive; anus opening subterminally. Two paired testes, discharging through paired gonoducts into a common midventral furrow. Paired ovaries, female pore unpaired, surrounded by a ventral epidermal glandular field (pseudo-clitellum). Seminal receptacle unpaired, opening just in front of female pore. No asexual reproduction. Mostly recorded from the bottom of large rivers, but also found on the shore of the Baltic Sea and, in large numbers, in the oligotrophic Lake Stechlin, Germany (Collado et al. 1999).

**Class** POLYCHAETA

**Family Nerillidae**

A family of minute worms endowed with small cephalic palps and antennae and cirri along the body and on the pygidium. They comprise 17 genera and about 48 species, mostly marine interstitial, occurring worldwide from the intertidal to abyssal depths (Worsaae and Kristensen 2005). One species, *Troglochaetus beranecki* Delachaux, 1921, has been found exclusively in subterranean freshwater habitats (phreatic and hyporheic sands and pebbles, caves, wells and springs), even at high altitude and in formerly glaciated areas, across a vast area of Europe (from Finland to the Alps, see Särkkä and Mäkelä 1998, and from France to Romania) and (with conspecific populations?) at several locations of the USA (Colorado, Montana, Virginia, Alabama, New Hampshire, Pennsylvania, Ohio; Strayer 2001 and Smithsonian Museum Collection). It is believed that its ancestors were members of psammon in epicontinental seas, from which the species, possibly before continental drift was well under way, entered the groundwater system.

**Family Parergodrilidae**

This family of unclear phylogenetic position comprises only two species: the marine littoral mesopsammic *Stygocapitella subterranea* and the truly terrestrial *Parergodrilus heideri* (Rota et al. 2001​). The latter inhabits the litter and organic soil horizons of a variety of inland woodlands (beech, conifer, holm oak) and occasionally, waterlogged habitats (Rota et al. 2010). Until recently *P. heideri* appeared restricted to Europe, but findings in the wild have been reported from Korea (Dózsa-Farkas and Hong 2010) and the USA (Minnesota and Wisconsin; Schlaghamerský and Frelich 2012). The possibility of the Parergodrilidae being close to the Orbiniidae has been proposed in some recent molecular analyses, but support was significant only in two such studies (Bleidorn et al. 2003, Struck et al. 2008).

**Family Serpulidae**

A worldwide-distributed family of polychaetes building tubes of calcium carbonate, comprising about 350 species, nearly all marine. *Marifugia cavatica* Absolon & Hrabě, 1930 is the sole member living in fresh water and is a stygobiont, endemic to the European alpine-dinaric karst. Molecular phylogenetics places *M. cavatica* as sister taxon to a clade of brackish-water *Ficopotamus* species, suggesting that the transition to the subterranean environment occurred via ancestral marine shallow water to intertidal or estuarine species (Kupriyanova et al. 2009). From intense cave biology research in Croatia, *Marifugia* is capable of withstanding prolonged periods outside the water but was never recorded in brackish conditions (Branko et al. 2012).

**Polychaeta*incertae sedis***

*Hrabeiella periglandulata* Pižl & Chalupský, 1984 is, along with the parergodrilid *Parergodrilus heideri* Reisinger, 1925, the sole truly terrestrial non-clitellate annelid (Rota 1998, Rota et al. 2001). It has been collected in woodland and grassland sandy soils throughout the European continent, from Spain to Romania and from Italy to Sweden, and has been recently reported from Korea (Dózsa-Farkas and Hong 2010). Its phylogenetic position is still enigmatic and since originally described, the taxon has remained unassigned to family. In the Fauna Europaea database it has been placed in the Parergodrilidae for merely database purposes, with such position corroborated neither by morphological nor by molecular data (Rota 1998, Rota et al. 2001).

**Part 2: Oligochaeta**

Class OLIGOCHAETA

**Family Acanthodrilidae**

Allochthonous megadrile family with a very wide geographic distribution, most probably non monophyletic. Endemic taxa can be found throughout the southern continents, plus North and Central America (Buckley et al. 2011). The male reproductive system includes primarily one or two paired prostates, opening separately on segment 17 and/or 19, while the single pair of male pores is generally located on 18 (acanthodriline arrangement). In the taxa showing a microscolecine reduction, the posterior prostates have disappeared and the male pores tend to be found close to the prostate pores in 17. There are sexprostatic species as well (i.e. prostates in 17, 18, 19) or the prostates can be shifted backward (as in many *Diplocardia* species). Each of the acanthodriline/microscolecine/balantine conditions involves not only the position of the prostate pores but also the position of the spermathecae, which are strictly connected. The microscolecine condition, combined with a weak gizzard and vesiculated nephridia, is observed in *Microscolex*, a genus with endemics in South America, South Africa and New Zealand. Two species, *M. dubius* and *M. phosphoreus*, possibly native to South America, are synanthropic and have been introduced worldwide. The former has been recorded in Europe only from countries bordering the Mediterranean (Portugal, Spain, France, Italy, Albania and Greece). The latter, notable for its bioluminescence, has shown a larger invasive capacity and adaptability: in Poland it has been found in high abundance in coal mines at 230 m below the surface, and in central Hungary the species was able to survive outdoor winter temperatures of -20°C (Skowron 1928, Csuzdi 1986; see Rota 2009).

**Family Ailoscolecidae**

Monotypic megadrile family endemic to southwestern France. They were regarded as possibly close to the North American Komarekionidae (Sims 1980, Qiu and Bouché 1998c), particularly because of having the male pores in segment 22 and prostate-like glands associated with the tubercula pubertatis. However, molecular evidence excludes any such kinship and places the deep-soil dwelling *Ailoscolex lacteospumosus* Bouché, 1969 within the Hormogastridae (James and Davidson 2012).

**Family Criodrilidae**

Monotypic megadrile family indigenous to the western Palaearctic. Primitively aquatic, *Criodrilus lacuum* Hoffmeister, 1845 is characterized by a quadrangular body section, the lack of gizzards and other gut specializations, the long clitellum, absence of spermathecae and tubercula pubertatis, and the production of long, horn-shaped spermatophores and long cocoons. Molecular data place it as sister taxon to the Hormogastridae and Lumbricidae (James and Davidson 2012). Although frequently parthenogenetic and capable of regeneration, as well as of living in aquaria for decades, it has never been recorded in the British Isles and Scandinavia and has only occasionally been introduced to other continents (e.g. the Americas; arrived in Baltimore, Maryland before 1900, McKey-Fender and MacNab 1953; recorded in Brazil rice fields, Knäpper and Porto 1979). Widely distributed in Europe and common all around the Mediterranean, its physiology and behaviour were first made popular by Lazzaro Spallanzani (Rota in prep.). Records appear nowadays scantier due to climate and habitat changes. Molecular taxonomy should ascertain whether, given the broad geographic distribution, some populations have achieved the species or subspecies status (*C. ochridensis* Georgevitch, 1950 from Lake Ochrid, Macedonia, is recorded in the FaEu database as a separate species).

**Family Enchytraeidae**

Microdrile family with worldwide distribution, Polar regions included. The Enchytraeidae are well separated from all other oligochaetes by a set of somatic (chaetae short and simple-pointed, emerging from a generally smooth body wall; prostomium pierced by a coelomic pore; pharyngeal glands as conspicuous masses arranged pairwise in segments 4-6, with ventral strands of ducts directed to the roof of pharynx; holonephridia with postseptal loops more or less anastomosed to form canalized compact bodies; coelomocytes abundant) and reproductive features (spermathecae opening in segment 5; testes in 11, ovaries in 12, with respective paired pores in 12 and 13; preseptal sperm funnel glandular, opposite end of vas deferens generally surrounded by a copulatory cushion). So far, the more inclusive molecular phylogenetic analyses (e.g. Erséus et al. 2010, James and Davidson 2012) have not confirmed any of the classical or recent hypotheses relating these worms to other microdrile families (most notably the Propappidae) or to the megadriles. The history of lineages within the family is also far from resolved, but some relationships appear well supported (Erséus et al. 2010), e.g. the 'achaetine' genera (sensu Černosvitov 1937) stemming out basally from the rest of the family, or the three genera possessing two types of coelomocytes (*Fridericia*, *Hemifridericia* and *Buchholzia*) forming a monophyletic clade.

**Figure 1.**
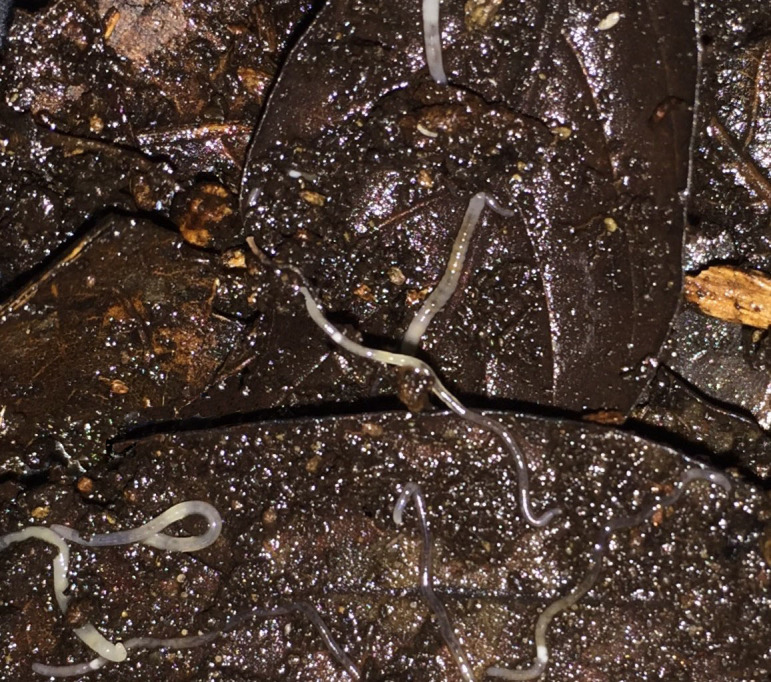
Enchytraeid species crawling through *Quercus ilex* leaf litter.

The vernacular name of the family, pot-worms (fr. Greek '*en*-*chytra*' = in flowerpot), does not reflect the great ecological diversity of the group, which, although most represented in terrestrial soils (Fig. 1), has also colonized freshwater and marine deposits (littoral, sublittoral and profundal), acid boglands, the snow-cover and the ice of glaciers, sewage beds, and other habitats where moisture conditions and food supply are suitable. Due to their sensitivity to hypoxia and desiccation, enchytraeid populations reach their greatest abundance and biomass in cold to temperate moist climates. Moreover, because these worms have no vascularization of the body wall, oversized species (> 30 by 2-3 mm) are confined at latitudes where oxygen availability rarely becomes a limiting factor, while moisture and nutrients are always abundant (Rota 2001). Nevertheless, many species tolerate low soil moisture during short periods and taxonomic richness, if properly investigated, can be equally high in hot (e.g. in Mediterranean forests, see Rota et al. 2014b; or in the Mata Atlantica of Brazil, see Römbke et al. 2005) as in cold climates. Currently over 700 species, classified in about 30 genera, are regarded as valid in the world (Schmelz and Collado 2015), but the global inventory is very far from complete.

About half of the currently accepted inland species have their type localities in Europe, where collecting and taxonomic work has always been comparatively intense. The northernmost latitudes of the Holarctic, particularly Beringia (refs. in Christensen and Dózsa-Farkas 2005), as well as certain regions and habitats of Brazil (refs. in Christoffersen 2009, Schmelz et al. 2013) follow, in terms of species discoveries. In the rest of the New World, a diverse fauna is documented for the forests and glaciers on the North Pacific coast (refs. in Schlaghamerský 2014), and two peculiar life forms appear exclusive to the southeastern US (the freshwater *Barbidrilus paucisetus* Loden & Locy, 1981) and the Caribbean (the epizoic *Pelmatodrilus planariformis* Moore, 1943), but huge areas of both Americas remain completely unexplored. Likewise, we have only hints of the probably high endemicity and diversity of tropical and temperate Africa, as well as of southern and eastern Asia and Australasia, and recent surveys in China (refs. in Wang and Cui 2007, Lian et al. 2011) and Korea (Dózsa-Farkas and Hong 2010, Christensen and Dózsa-Farkas 2012, Dózsa-Farkas et al. 2015) point to complex biogeographical relationships among the major world regions. The geographical sampling bias is only one of the reasons for the incompleteness of the global inventory. The number of taxonomists working on this ubiquitous family has always been low, both because of practical difficulties in sampling, and because species identification and differentiation traditionally involve the observation of live specimens (marine forms are generally less difficult to identify in a preserved state).

The correct evaluation of species taxonomic status and distribution is a fundamental prerequisite in assessing biodiversity in any geographical region. Both these aspects are still in a state of uncertainty for many European components of the family. According to the published records, a fair number of species would appear Holarctic or even cosmopolitan. However, besides the mentioned sampling biases, many records date back to a time when identifications were based on either ambiguous diagnoses and/or inadequate examinations. Since the mid-1990s, there has been an effort to better characterize the species through an enlarged and univocal set of features (e.g. Rota 1994a, Rota 1994b, Rota and Healy 1994, Rota 1995, Rota et al. 1998, Rota and Healy 1999, Schmelz 2003, Schmelz and Collado 2010, Rota 2013b), ultimately implying the combined use of living and fixed material (e.g. Rota and Healy 1999, Schmelz 2003, Rota 2013b, Rota 2015). This effort has clarified the identity of several common species previously regarded as extremely variable, and has provided a foundation, often on a continental scale, for subsequent studies of species diversity and endemicity. Furthermore, in the last decade the application of molecular methods to species delimitation has started uncovering some cryptic species (e.g. Cech and Dózsa-Farkas 2005, Martinsson and Erséus 2014, Martinsson et al. 2014, Martinsson et al. 2015, Dózsa-Farkas and Felföldi 2015), while allowing the re-evaluation of the status of some taxa currently treated as distinct species (e.g. Cech et al. 2012, Dózsa-Farkas et al. 2012). In the first version of the Fauna Europaea database in 2003, the family was represented by 233 species in 19 genera. Since then, some 40 new species have been described from our continent.

**Family Eudrilidae**

The Eudrilidae, a megadrile family of tropical West and East Africa, are separated from all other earthworms by their specialized “spermathecal systems”, which are found posterior to the testis segments and are connected to the oviducts. One species, *Eudrilus eugeniae*, known in the fish bait market as the “African night crawler”, is a native to West Africa that has become pantropical at low altitudes. It is a relatively large earthworm that under constant high temperatures (25°–30°C) grows extremely rapidly, is prolific and thus exploited for protein production, composting and agriculture. Its main disadvantages are sensitivity to handling and its narrow temperature tolerance (it grows very slowly at 15°C and dies below 5°C; Domínguez et al. 2001, Domínguez and Edwards 2011). Outdoor vermiculture is therefore limited to tropical and subtropical regions, while in temperate regions the species can only survive in greenhouses. Beddard (1906) recorded it in Kew from soil in a Wardian case brought from British Guiana (Guyana), and Csuzdi et al. (2008) found it in a tropical plant nursery in Hungary. No other records are known from Europe, except for laboratory cultures (Spain, Germany).

**Family Glossoscolecidae**

Megadrile earthworms indigenous to the tropical forests of South and Central America, with clitellum beginning near segment 14, endowed with tubercula pubertatis. Male pores either inconspicuous or within copulatory chambers. Dorsal pores lacking, oesophageal gizzard in segment 6; extramural calciferous glands in some or all of segments 7-14; typhlosole present. Holoic with nephridial bladders in intestinal region. Spermathecae adiverticulate, in front of the gonadal segments. The family in this classical acception harbours 200 species and 25 genera, but molecular phylogeny has recently revealed it as polyphyletic (James and Davidson 2012), consisting of a Glossoscolecidae sensu stricto clade (*Glossoscolex*, *Glossodrilus*, *Righiodrilus*, *Fimoscolex* etc., morphologically sharing paired calciferous glands at 11/12, male pores conspicuous and usually with muscular ejaculatory bulbs, typhlosole consisting of a compact lamina with a complex folding) and a reestablished Rhinodrilidae Benham, 1890 comprising all other genera (James 2012). Type species of the latter is the endogeic *Pontoscolex corethrurus*, which has been transported widely by man and has colonized most disturbed soils in the tropics (Lavelle et al. 1987). In Europe it has been recorded in greenhouses in Germany, Finland and the UK. The only record around the Mediterranean is by Michaelsen (1938), from a thermal spring locality in northern Algeria (Hamman Righa, material collected by F.E. Beddard).

**Family Hormogastridae**

Megadrile family endemic to the western Mediterranean region, whose distinctness from the common European earthworms (Lumbricidae) was first recognized by Francesco Redi (Rota 2011​). They presently comprise four genera (*Hormogaster* Rosa, 1887, *Hemigastrodrilus* Bouché, 1970, *Vignysa* Bouché, 1970, *Xana* Díaz-Cosín et al. 1989) and 30 nominal species and subspecies. Their close affinities with Lumbricidae, Ailoscolecidae, Criodrilidae and Lutodrilidae (to form the Lumbricoidea s.s.) to the South African Microchaetidae (Omodeo 2000) is supported by molecular phylogeny (James and Davidson 2012). The Hormogastridae are characterized by the lack of dorsal coelomic pores, the possession of two or three anterior (postpharyngeal) gizzards, and the clitellum beginning just before the male openings, which are located near 15/16. Body size in these worms, particularly in the genus *Hormogaster*, is mostly large or even very large, reaching up to 90 cm in length and 100 g in weight (Omodeo and Rota 2008). Because of their endogeic habits, physiological adaptation to prolonged periods of drought (thanks to a long diapause), and low vagility, the Hormogastridae have often being the subject of biogeographical studies addressing the complex climatic history and land evolution of the western Mediterranean (Omodeo 1956, Bouché 1972, Bouché 1983, Omodeo 1984, Omodeo and Rota 1987, Cobolli Sbordoni et al. 1992, Qiu and Bouché 1998b, Omodeo and Rota 2008, Novo et al. 2011, Novo et al. 2014). The phylogenetic relationships within the family are hardly recognized being generally hampered by inconsistent patterns of morphological differentiation (e.g. Novo et al. 2012a). Molecular studies (Cobolli Sbordoni et al. 1992, Novo et al. 2009, Novo et al. 2010, Novo et al. 2011, Novo et al. 2012b, Novo et al. 2014) have also pointed out the occurrence of highly divergent cryptic lineages within certain morphospecies (e.g. *H. pretiosa*, *H. elisae*), despite the low geographical distance between populations, and a much lower genetic differentiation within and between other morphospecies separated by a long geographical distance. Interestingly, the biogeographical analysis carried out by Novo et al. 2014 on the easternmost areas of the family's range has shown a correspondence between the patterns of diversification of the individual hormogastrid species and the archipelago-like relationships between the earthworm faunal assemblages of Tyrrhenian districts formerly illustrated by Omodeo and Rota (1987), Rota (1992), Omodeo and Rota (2008). Furthermore, *H. elisae* appears as a relatively independent monophyletic species complex occupying a restricted area in central Spain (Novo et al. 2014), whilst the Sardinian-Franco-Iberian morphospecies *H. pretiosa* (sensu Cobolli Sbordoni et al. 1992) has turned out to be polyphyletic, with the French populations (= *H. gallica* Rota, 1994) belonging to a separate clade, which justifies the high values of allozymic divergence measured by Cobolli Sbordoni et al. (1992). The high diversity of species of *Hormogaster* in the southern Pyrenées, ventured by Qiu and Bouché (1998a) on the basis of not always clearcut morphological evidence, was dismissed as "*H. pretiosa* species complex" in the previous version of FaEu database, with the taxonomic comment: "Qiu and Bouché (1998a) recognized as many as 11 separate new species in material from Southern Pyrenées (*H. riojana*, *H. ireguana*, *H. eserana*, *H. huescana*, *H. lleidana*, *H. multilamella*, *H. arenicola*, *H. catalaunensis*, *H. sylvestris*, *H. najaformis*, *H. castillana*)". However, DNA sequence data from multiple markers (Novo et al. 2011, Novo et al. 2012b) confirm the genetic diversification and taxonomic validity for most of those species. The discovery of *H. joseantonioi* Fernández-Marchán et al. 2014 in Aragón, and its placement as sister taxon of *H. elisae*, further supports the anticipation that several representatives of this family await discovery in unexplored regions of suitable habitat.

**Family Lumbricidae**

**Figure 2.**
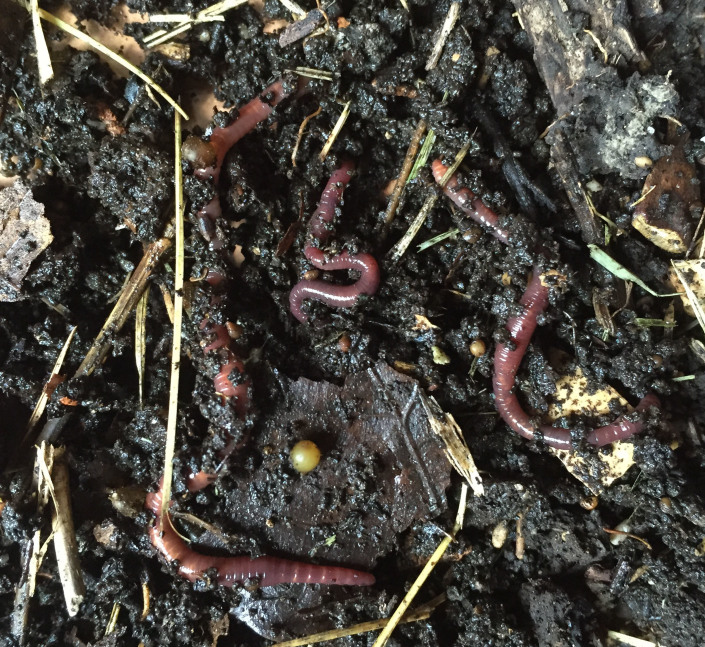
Epigeic lumbricid earthworms and their cocoons.

**Figure 3.**
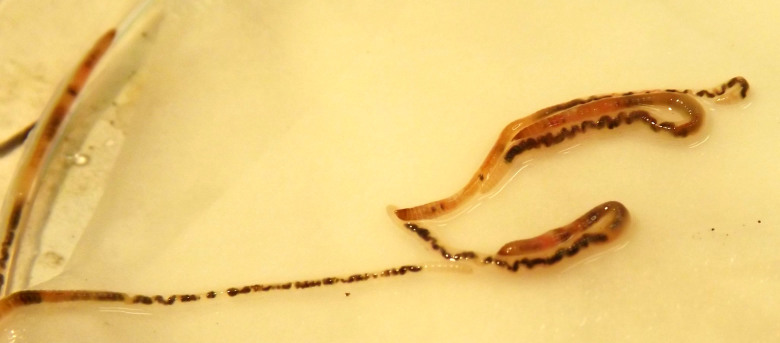
Specimens of *Dendrobaena cognettii* (Michaelsen, 1903), one of the smallest European earthworms (Lumbricidae), crawling around in a Petri dish.

Megadrile family endemic to the Holarctic (Figs 2, 3), occurring naturally from the central USA to central Asia, with its highest diversification in Europe where it comprises at least 450 valid species. Major hotspots of endemism are located in the Franco-Iberian area, the Carpathian Basin, the Caucaso-Anatolian area. The family is diagnosed by having the clitellum starting never before segment 19 and the stomach and gizzard lying behind the oesophagus in 15-16 and 17-18 (or more), respectively; coelomic pores open middorsally (dorsolaterally paired in Diporodrilinae), the tubercula pubertatis are located before (Spermophorodrilinae) or within the clitellum (Lumbricinae and Diporodrilinae), and the sexual pores open laterally between chaetae *b* and *c* (Lumbricinae and Diporodrilinae), or ventrally near chaeta *b* or between chaetae *a* and *b* (Spermophorodrilinae).

The Lumbricidae are most closely related to the Hormogastridae, Ailoscolecidae, Lutodrilidae and Criodrilidae, all families endemic to our continent (James and Davidson 2012).

In the first version of the Fauna Europaea database in 2004, the family Lumbricidae was represented by 384 species classified in 32 genera and the three subfamilies mentioned above, following criteria that took into account classical views and well-grounded modern morpho-taxonomy. Molecular cladistic analyses focusing on earthworms had then just started and for about a decade phylogenetic reconstructions of the Lumbricidae would be hindered by restricted taxonomic sampling and/or the low signal of the chosen genes (Pop et al. 2003, Cech et al. 2005, Pop et al. 2005, Pop et al. 2007, Briones et al. 2009, Pérez-Losada et al. 2012). In any case, albeit limited (see review by Chang and James 2011), those studies repeatedly called attention to the heterogeneity of several genera (*Allolobophora*, *Dendrobaena*, *Aporrectodea*, *Eisenia*) as diagnosed by most authors on a morphological basis. More recently, using DNA data from multiple markers (with different evolutionary rates) and an extensive taxonomic sampling, Domínguez et al. (2015) have provided a comprehensive and quite robust molecular phylogeny of the family that (except for some nomenclatural problems and possible misidentifications) suggests the urgent need for reevaluation of many widely used lumbricid genera in an integrative taxonomic framework. Looking at the topology of the molecular tree, not only the homoplasy of basic morphological traits, but also the inconsistent evolutionary pattern of more sophisticated anatomical criteria used in earthworm taxonomy (e.g. the type of body wall musculature, the shape and orientation of the nephridial bladders, etc.) appear evident. Domínguez et al. (2015) also give solid evidence of the multiple origins of reproductive, feeding and burrowing habits in the family, as well as of the correlations between genealogical lineages and geographical distributions (consequence of a generally low dispersal capability). DNA sequence data is in sum essential for lumbricid phylogenetic reconstructions, but using an extended set of non trivial morphological traits (including the structure of the modified genital chaetae) plus geographical information, appears crucial for recircumscriptions and characterizations of genera and higher-level relationships in the family.

At the higher levels, while the molecular data in Domínguez et al. (2015) confirm the classification of the Sardo-Corsican genus *Diporodrilus* in a distinct subfamily (deserving full family rank acc. to Bouché 1970), they seem to dismiss the Balkan-Anatolian Spermophorodrilinae Omodeo & Rota, 1989 (by denying the monophyly of *Healyella*), an issue, however, that remains pending, due to limited sampling and the non-inclusion of the type genus and species. Thus the Spermophorodrilinae is maintained in the present updating of the FaEu database. Likewise, the molecular analyses of Domínguez et al. (2015) support the monophyly of the genera *Postandrilus* (Balearic species), *Eiseniona*, *Eisenia*, *Lumbricus*, *Prosellodrilus*, and *Scherotheca*, and suggest the para- or polyphyly of *Allolobophora*, *Aporrectodea*, *Cataladrilus*, *Cernosvitovia*, *Dendrobaena*, *Healyella*, *Helodrilus*, *Octodrilus* and *Octolasion*, but a formal evaluation of the taxonomic validity of all these genera will require inclusion of the respective generotype. Interestingly, the strongly supported clade comprising *Eisenia* (sensu stricto), *Eisenoides*, *Allolobophoridella*, *Dendrodrilus* and *Bimastos* may find morphological evidence in the number of longitudinal grooves (3 instead of 4) characterizing the genital chaetae of these genera (Kvavadze 2000), but the nominal species of *Dendrobaena* also sharing this character (*byblica, ganglbaueri, alpina*), allocated by Kvavadze (1993), Kvavadze et al. (2007) to the genus *Omodeoia*, should then have acquired this character independently. Interestingly also, the western archaic genus *Prosellodrilus* occupies with *Postandrilus* and other Iberian species of problematic affiliation a basal position, as does *Eophila* as redescribed by Omodeo and Rota (2004) (the latter would seem to pass the monophyly test, even though the type of the genus (*Eo. tellinii*) was not involved in the analysis by Domínguez et al. (2015) and although the relationships with certain nominal species of *Cernosvitovia* should be reconsidered).

**Family Megascolecidae**

Allochthonous megadrile family, very widely distributed, whose most ancient taxa show a predominant southeastern distribution (Australia, New Zealand, southeastern Asia), but with a tribe, the Argilophilini, endemic to western North America (Fender 1995). Chaetal arrangement lumbricine or perichaetine. Penial chaetae mostly absent. Meroic and/or holoic nephridia, some taxa enteronephric. One to six paired spermathecae. Prostates one pair, tubular or racemose, opening united with vasa deferentia in segment 18 (megascolecine arrangement). Several genera regarded as native to southeastern Asia (e.g. *Amynthas*, *Metaphire*, *Perionyx*, *Pithemera*, *Pontodrilus*, *Spenceriella*) have species distributed worldwide in tropical and temperate regions, both in natural and cultivated areas. In Europe they are mostly found in artificial environments such as greenhouses, botanical gardens, urban greens and exotic plantations.

**Family Ocnerodrilidae**

Allochthonous family of small, filiform, semiaquatic megadriles, sister taxon to the Megascolecidae s.l. (Jamieson et al. 2002, James and Davidson 2012; but see Rota 2013a for a summary of different phylogenetic opinions). All of the endemic genera and species are confined to South and Central America, sub-Saharian Africa, and India but several peregrine species are currently distributed at tropical and subtropical latitudes worldwide. In Europe, early authors documented the arrival of several distinct taxa in botanical gardens, but outdoor records were until not so long ago restricted to collections of *Ocnerodrilus occidentalis* and *Eukerria saltensis* in southern countries (Spain, Portugal and Italy; Szederjesi 2015 also recorded *O. occidentalis* in a pine forest in Greece). Rota (2013a) has recently reported several new outdoor finds of these two species from Corsica up to the Thames in central London (51°30’N) and suggested that the trade of ornamentals and horticultural products as well as current environmental and climate changes are responsible for a substantial increase of their dispersal and survival possibilities, which may cause an accelerated spread of them through the continent.

**Family Octochaetidae**

The earthworm genus *Dichogaster* is a large, heterogeneous taxon comprising some 350 species allocated to three subgenera (Csuzdi 2014). It is diagnosed by presence of penial chaetae, an acanthodriline condition of the male apparatus (two paired prostates, opening separately on segments 17 and 19, and a single pair of male pores on 18; as opposed to the megascolecine condition where one pair of male and prostatic pores unite on 18), meroic excretory system (more than two nephridia per segment), two gizzards in 6-7 and three pairs of calciferous glands in the region of 15–17. *Dichogaster* belongs with other megascolecoid genera to subfamily Benhamiinae, but family level classification is controversial. With regard to the nephridial system, according to Csuzdi 1995, Csuzdi 1996, *Dichogaster* would be a deviant representative of holoic Acanthodrilidae, whereas according to Blakemore (2005), *Dichogaster* belongs to separate meroic Octochaetidae. However, neither of these two classical morphology-based families is supported by molecular data (James and Davidson 2012), which rather consider *Dichogaster* and the other Benhamiinae as a specialized clade within Megascolecidae s.l. Octochaetidae were regarded as valid in the earlier version of the FaEu database. Since there is still no agreement on the familial position of Benhamiinae, we prefer to maintain here the previous classification.

Endemic species of *Dichogaster* are restricted to Central and South America and Africa, but some congeners have spread synanthropically in most parts of the world. Particularly two of them, *D. bolaui* and *D. saliens*, are among the most frequent peregrine species in tropical and subtropical regions. The prominent vascularization of their body wall and inner organs facilitates their survival and spreading under flooded conditions. According to Csuzdi (1995), these species belong to the subgenus *Diplothecodrilus* Csuzdi, 1996, which is probably native to tropical eastern Africa. In the last decades, both these earthworms have shown their ability to thrive and spread within the plumbing systems of urban buildings in Fennoscandia (Terhivuo 1991, Erséus et al. 1994). Once colonized, possibly by escapees from ornamental potted plants, the sewer system provides a buffered microclimate and constant food supply (biofilm, organic residues), and acts as a secondary dispersal source sheltered from the rigours of the outdoor climate in cities as far north as Oulu in Finland. More recent findings of *D. bolaui* in the same type of habitat in Hungary (Csuzdi et al. 2008) and in a heated swimming pool in Cork, Ireland (Rota and Schmidt 2006) document how the same process of colonization could be under way in other parts of the Old World.

**Family Propappidae**

Monogeneric microdrile family, endemic to the Palaearctic region. Although fully aquatic, it is dealt with in this paper because of its historical taxonomic association with the Enchytraeidae. The first *Propappus* species (*P. glandulosus* Michaelsen, 1905) was discovered in Lake Baikal and placed in the Enchytraeidae, to represent an early branch within the family. It would be later recorded also in surrounding rivers in Russia (Čekanovskaya 1962). The second species, *P. volki* Michaelsen, 1916, originally described from the Elbe River, Germany, appears distributed from western Europe through central Russia and the Russian Far East and Japan (Bird 1982, Coates 1986, Torii 2006, Timm 2009) and has also been recorded in the St. Lawrence River in Canada (K.A. Coates, in Torii 2006). A third species, *P. arhyncotus* Sokolskaya, 1972, first described from the Kamchatka Peninsula, has been collected also in the Amur basin in the farthest southeast of Russia (Timm 1994, Timm 1999). *Propappus* differs substantially from the Enchytraeidae, both morphologically and genetically. Its species have: sigmoid, nodulate, bifid chaetae; large epidermal glands opening through the body wall posterior to each chaetal bundle; spermathecal pores posterior to septum 3/4; glandular part of vasa deferentia located posterior to septum 11/12; and one pair of ovaries in segment 13 with female funnels located on septum 13/14. Based on this evidence, Coates (1986) removed *Propappus* from Enchytraeidae to form a separate monotypic family, the Propappidae. According to Brinkhurst (1994), the two families would be sister taxa; however, a comprehensive molecular analysis has failed to recover any close relationship between them (Erséus et al. 2010). Ovary organization and oogenesis are also stated to differ between the two families (Gorgoń et al. 2015), but the comparison should be extended to include more representatives of enchytraeids (for instance, Rota and Manconi 2004 reported different types of ovary organization among enchytraeid species of *Marionina*).

The only propappid species occurring in Europe, the proboscidate, interstitial *P. volki*, appears to be a good bioindicator for unpolluted water (Torii 2006).

**Family Sparganophilidae**

Megadrile family of very slender worms, measuring as adults 70-200 mm in length and 2-3.5 mm in maximal width, spending their life in the mud or amongst the roots of aquatic plants. The family comprises one genus and about a dozen species, nearly all restricted to the southeastern USA. The type species, *Sparganophilus tamesis* Benham, 1892, originally discovered in River Thames, England (Benham 1892), is itself native to eastern North America and very widespread there (from southeastern Canada to the Gulf States, plus isolated collections in Mexico and Guatemala; Reynolds 2008 as *S. eiseni* Smith, 1895). Another two species appear endemic to California and Oregon, and immature sparganophilids have been reported from a swampy forest in Guyana (Stacey and Coates 1996, Reynolds 2008). The American origin of Sparganophilidae is confirmed by molecular phylogenetics (Jamieson et al. 2002, James and Davidson 2012), which places the family as sister taxon to Komarekionidae, a monotypic earthworm family living in mesic forest soils on the Appalachian Mountains (Rota et al. 2014a).

Besides England, the occurrence of the family outside of America is limited to France, Switzerland, Germany, Italy (Rota et al. 2014a). Many early records of *Sparganophilus*, both in Europe and America, were originally assigned to distinct species. In the light of the intrapopulational morphological variability documented in *S. tamesis*, very likely all European records are conspecific (Jamieson 1971, Rota et al. 2014a​). The vector for the arrival of *Sparganophilus* worms to European waters were most certainly North American aquatic plants accidentally introduced. The synergistic interaction between submerged plants (such as *Vallisneria*) and *Sparganophilus* worms has been recently pointed out, as well as the possible reciprocal importance for the survival and spreading of the two organisms even over long distances and particularly under the ongoing climate changes (Rota et al. 2014a​).

## Project description

### Title

This BDJ data paper includes and updates the taxonomic indexing efforts in the Fauna Europaea on European Annelida-Oligochaeta (terrestrial: Enchytraeidae and Megadrili), Aphanoneura and Polychaeta covering the first two versions of Fauna Europaea worked on between 2000 and 2013 (up to version 2.6).

### Personnel

The taxonomic framework of Fauna Europaea includes partner institutes, providing taxonomic expertise and information, and expert networks taking care of data collation.

Every taxonomic group is covered by at least one Group Coordinator responsible for the supervision and integrated input of taxonomic and distributional data of a particular group. The Fauna Europaea checklist would not have reached its current level of completion without the input from several groups of specialists. The formal responsibility of collating and delivering the data of relevant families rested with a number of Taxonomic Specialists (see Table 1). For Annelida-Oligochaeta (terrestrial: Enchytraeidae and Megadrili), Aphanoneura and Polychaeta, the responsible Group Coordinator and Taxonomic specialist is Emilia Rota. A more detailed overview of the Fauna Europaea classification and expertise network for Annelida-Oligochaeta (terrestrial) can be found here: http://www.faunaeur.org/experts.php?id=101.

Data management tasks are taken care of by the Fauna Europaea project bureau. During the project phase (until 2004) a network of principal partners took care about diverse management tasks: Zoological Museum Amsterdam (general management & system development), Zoological Museum of Copenhagen (data collation), National Museum of Natural History in Paris (data validation) and Museum and Institute of Zoology in Warsaw (NAS extension). Since the formal project ending (2004-2013) all tasks have been undertaken by the Zoological Museum Amsterdam. Since 2013 the data servers are hosted at the Museum für Naturkunde in Berlin (migrated from ZMA-UvA).

On the available expert capacity, presently, in Europe faunistic, systematic and taxonomic studies on terrestrial Annelida (clitellate and non-clitellate) species are actively carried out in Italy (University of Siena), Hungary (Eötvös Loránd University; Eszterházy Károly College), Germany (IFAB Institut für Angewandte Bodenbiologie; ECT Oekotoxikologie; Senckenberg Research Center for Biodiversity and Climate), Denmark (University of Copenhagen), Spain (Universidad Complutense de Madrid, Universidade da Coruña, University of Vigo), Romania (Institute of Biological Research), Czech Republic (Masaryk University), Poland (Polish Academy of Sciences), UK (Natural History Museum, London; University of Cardiff), Sweden (University of Göteborg) and in Ireland (University College Dublin), by around 20 specialists, often with expertise in one or two familes. Megadriles and Enchytraeidae are generally investigated by separate communities, the one involved in enchytraeid alpha-taxonomy being composed of around six experts in Europe (and world-wide). Additional ultrastructural research is carried out in Poland (University of Silesia) and Germany (University of Osnabrück). Outside Europe around ten more specialists contribute to the taxonomy of terrestrial (clitellate and non-clitellate) Annelida, and phylogenetic studies are presently mostly conducted as intercontinental collaborations (often with North American laboratories: University of Guelph, Canada; University of Iowa; University of Washington; Harvard University; US National Museum of Natural History, Smithsonian Institution).

### Study area description

The area study covers the European mainland (Western Palearctic), including the Macaronesian islands, excluding the Caucasus, Turkey, Arabian Peninsula and Northern Africa (see: Geographic coverage section).

### Design description

*Standards*. Group coordinators and taxonomic specialists have to deliver the (sub)species names according to strict standards. The names provided by Fauna Europaea are scientific names. The taxonomic scope includes issues like, (1) the definition of criteria used to identify the accepted species-group taxa, (2) the hierarchy (classification scheme) for the accommodation of all the accepted species and (3), relevant synonyms, and (4) the correct nomenclature. The Fauna Europaea 'Guidelines for Group Coordinators and Taxonomic Specialists', include the standards, protocols, scope, and limits that provide the instructions for more than 400 specialists contributing to the project, strictly following the provisions of the current edition of the International Code of Zoological Nomenclature.

*Data management*. The data records could either be entered offline into a preformatted MS-Excel worksheet or directly into the Fauna Europaea transaction database using an online browser interface. The data servers were hosted at the University of Amsterdam (Amsterdam academic computing facilities) until 2013, when hosting was migrated to the Museum für Naturkunde in Berlin.

*Data set*. The Fauna Europaea basic data set consists of: accepted (sub)species names (including authorship), synonym names (including authorship), a taxonomic hierarchy/classification, misapplied names (including misspellings and alternative taxonomic views), homonym annotations, expert details, European distribution (at country level), Global distribution (only for European species), taxonomic reference (optional), occurrence reference (optional).

### Funding

*Fauna Europaea* was funded by the European Commission under the Fifth Framework Programme and contributed to the Support for Research Infrastructures work programme with Thematic Priority Biodiversity (EVR1-1999-20001) for a period of four years (1 March 2000 - 1 March 2004), including a short 'NAS extension', allowing EU candidate accession countries to participate. Follow-up support was given by the EC-FP6 EDIT project (GCE 018340), by the EC-FP7 PESI project (RI-223806) and by the EC-FP7 ViBRANT project (RI-261532). Continuing management and hosting of the Fauna Europaea services was supported by the University of Amsterdam (Zoological Museum Amsterdam) and SARA/Vancis. Recently the continuation of Fauna Europaea was taken over by the Museum für Naturkunde in Berlin, supported by the EC-FP7 EU BON project (grant agreement №308454).

## Sampling methods

### Study extent

See spatial coverage and geographic coverage descriptions.

### Sampling description

Fauna Europaea data have been assembled by principal taxonomic experts, based on their individual expertise, including literature sources, collection research, and field observations. In total no less than 476 experts contributed taxonomic and/or faunistic information for Fauna Europaea. The vast majority of the experts are from Europe (including EU non-member states). As a unique feature, Fauna Europaea funds were set aside for rewarding/compensating for the work of taxonomic specialists and group coordinators.

**Figure 4.**
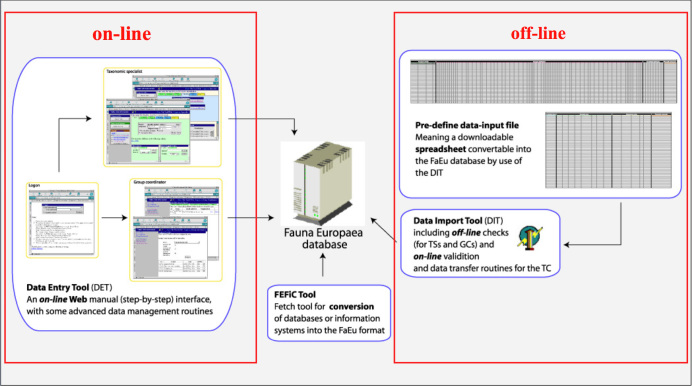
Fauna Europaea on-line (browser interfaces) and off-line (spreadsheets) data entry tools.

To facilitate data transfer and data import, sophisticated on-line (web interfaces) and off-line (spreadsheets) data-entry routines were built, integrated within an underlying central Fauna Europaea transaction database (Fig. 4). This includes advanced batch data import routines and utilities to display and monitor the data processing within the system. In retrospect, it seems that the off-line submission of data was probably the best for bulk import during the project phase, while the on-line tool was preferred to enter modifications in later versions. This system worked well untl 2013, but will be replaced by a new system in 2015.

A first release of the Fauna Europaea index via the web-portal had been presented at 27^th^ of September 2004, the most recent release (version 2.6.2) was launched on 29 August 2013. An overview of Fauna Europaea releases can be found here: http://www.faunaeur.org/about_fauna_versions.php.

### Quality control

Fauna Europaea data are unique in the sense that they are fully expert based. Selecting leading experts for all groups assured the systematic reliability and consistency of the Fauna Europaea data.

Furthermore, all Fauna Europaea data sets are intensively reviewed at regional and thematic validation meetings, at review sessions on taxonomic symposia (for some groups), by Fauna Europaea Focal Points (during the FaEu-NAS and PESI projects) and by various end-users sending annotations using the web form at the web-portal. Additional validation on gaps and correct spelling is being done by the validation office in Paris.

In general we expect to get taxonomic data for 99.3% of the known European fauna after the initial release. The faunistic coverage is not quite as good, but is nevertheless 90-95% of the total fauna. For terrestrial Annelida-Oligochaeta (terrestrial) the current taxonomic coverage is about 93% (see Table 1).

Gaps of knowledge in this group are difficult to quantify. For some families, the number of existing species has remained unchanged for years and only molecular taxonomy may alter it. With regard to the Enchytraeidae, Lumbricidae and Hormogastridae, not only barcoding analyses of common taxa continuously uncover distinct lineages which may represent cryptic species, but investigations conducted in insufficiently explored areas of our continent do not cease to provide evidence of new morphospecies.

Checks on technical and logical correctness of the data have been implemented in the data entry tools, including around 50 business rules. This validation tool proved to be of huge value for both the experts and project management, and contributed significantly to preparation of a remarkably clean and consistent data set. This thorough reviewing makes Fauna Europaea the most scrutinised data set in its domain.

To optimise the use and implementation of a uniform and correct nomenclature, a cross-referencing of the *Fauna Europaea* Annelida-Oligochaeta (terrestrial: Enchytraeidae and Megadrili), Aphanoneura and Polychaeta data-set with relevant nomenclators, including Nomenclatura Oligochaetologica, compiled by John W. Reynolds and Mark J. Wetzel, is recommended, following the global efforts on establishing 'ZooBank' as a component of a so-called 'Global Names Architecture' (Pyle and Michel 2008). A species list of world oligochaete families and freshwater Aphanoneura and Polychaeta, with synonyms, has been recently compiled by Dr. Tarmo Timm, with support from the Estonian Biodiversity project at the Nature Museum of Tartu University in Tartu, Estonia. Records of taxa included in that registry are based on specimens deposited in collections, environmental DNA, human observations, and references. The extra-European terrestrial earthworm families are not compiled at species level. URL addresses: http://elurikkus.ut.ee/elr.php?lang=est (in Estonian), and http://elurikkus.ut.ee/elr.php?lang=eng (in English). On the earthworm front, an accurate and useful online database of all families has been compiled by Csaba Csuzdi (http://earthworm.uw.hu/). This database can be searched by every field i.e. family, genus, specific epithet, author, year, reference to the original description and optionally the valid combination of the species name (author’s view) and deposition of type specimens (Csuzdi 2012).

### Step description

By evaluating team structure and life cycle procedures (data-entry, validation, updating, etc.), clear definitions of roles of users and user-groups, according to the taxonomic framework were established, including ownership and read and write privileges, and their changes during the project's life-cycle. In addition, guidelines on common data exchange formats and codes have been issued (see also the 'Guidelines for Experts' document).

## Geographic coverage

### Description

**Figure 5.**
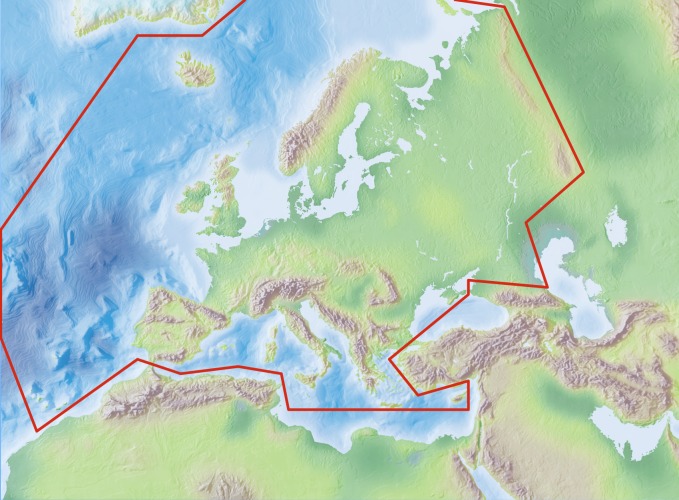
Fauna Europaea geographic coverage ('minimal Europe').

Species and subspecies distributions in Fauna Europaea are registered at least to country level, i.e. for political countries. For this purpose the FaEu geographical system basically follows the TDWG standards. The covered area includes the European mainland (Western Palearctic), plus the Macaronesian islands (excl. Cape Verde Islands), Cyprus, Franz Josef Land and Novaya Zemlya. Western Kazakhstan and the Caucasus are excluded (see Fig. 5).

The focus is on species (or subspecies) of European multicellular animals of terrestrial and freshwater environments. Species in brackish waters, occupying the marine/freshwater or marine/terrestrial transition zones, are generally excluded.

Additional notes and updating information on the geographic coverage of Annelida – Oligochaeta (terrestrial: Enchytraeidae and Megadrili), Aphanoneura and Polychaeta in Fauna Europaea can be found below:

**Family Aeolosomatidae**

On global coverage: Pinder (2004) reports *A. hemprichi* from Indonesia (ORR). Land (1970) reports (with doubts) *A. hyalinum* from Suriname (NEO). Wang and Cui (2007) report *A. headleyi*, *A. hemprichi*, *A. hyalinum*, *A. travancorense* from China (EPA). New records for Iraq (NRE) includes: *A. leidyi and A. quaternarium* (Al-Abbad 2012, Jaweir and Radhi 2013).

On European coverage: New records for Portugal includes (Picciochi 1982): *A. hemprichi*, *A. tenebrarum*, *A. travancorense*, *A. variegatum*. New records for Austria includes: *A. hyalinum*, *A. niveum* (Hörner et al. 1995). New records for Czech Republic includes: *A. headleyi*, *A. niveum*, *A. quaternarium*. New records for Slovak Republic includes: *A. hemprichi*, *A. quaternarium*, *H. chappuisi* (Schlaghamerský 2010). For *Hystricosoma chappuisi* there are new records for France (Route et al. 2004), Bulgaria (Subchev and Stamirinova 1986), Greece (Subchev et al. 2007), Austria (Berger et al. 2012). New records for Turkey include: *A. headleyi*, *A. tenebrarum*, *A. variegatum* (Balik et al. 2004).

**Family Potamodrilidae**

On global coverage: *Potamodrilus* sp. is observed in North America (Strayer 2001)

On European coverage: A new record for Turkey includes: *P. fluviatilis* (Balik et al. 2004).

**Family Nerillidae**

On global coverage: *T. beranecki* North American records: New Hampshire, Pennsylvania, Ohio (http://collections.mnh.si.edu/).

**Family Parergodrilidae**

On European and global coverage: *Parergodrilus heideri* new records includes: Denmark (Rota et al. 2010), Spain (Martinez-Ansemil and Parapar 2009), Korea (EPA) (Dózsa-Farkas and Hong 2010) and the USA (NEA) (Schlaghamerský and Frelich 2012).

**Polychaeta*incertae sedis***

On European and global coverage: *Hrabeiella periglandulata* new records: Austria, Spain, Denmark, Hungary, Romania (Dózsa-Farkas and Schlaghamerský 2013); Korea (EPA) (Dózsa-Farkas and Hong 2010).

**Family Acanthodrilidae**

On global coverage: new records: Japan (EPA38) (Blakemore 2012b), Jordan (Csuzdi and Pavlíček 2005), but already recorded from NRE.

**Family Enchytraeidae**

In the last 15 years, many new and known species of enchytraeids have been described in good detail. This effort and the sampling of new areas and environments have not only increased the length of the local inventories (for some countries the list has been almost doubled) but also improved their quality.

*First European records* include: *Enchytraeus luxuriosus* Schmelz & Collado, 1999 and *Hemifridericia bivesiculata* Christensen & Dózsa-Farkas, 2006. New regional records (since 2004) fall into all geographical units and are too substantial to detail here.

**Family Eudrilidae**

On European coverage: *Eudrilus eugeniae* new records: Hungary (Csuzdi et al. 2008).

**Family Glossoscolecidae**

On European coverage: *Pontoscolex corethrurus* new records: erroneously omitted for England GB-GRB (greenhouses at Kew Gardens; Sims and Gerard 1985, Sherlock and Carpenter 2009). Actually, "the commonest among earthworms accidentally brought to Kew Royal Gardens in Wardian cases" (Beddard 1894).

**Family Lumbricidae**

In addition to the newly discovered species, in the last 10 years single new records, checklists and biogeographical analyses have been produced for the lumbricid faunas of several countries (e.g. Portugal, Ireland, Great Britain, Germany, Hungary, Romania, Bulgaria, Croatia, Bosnia Herzegovina, Serbia, Montenegro, Macedonia, Albania, Greece) and mountain systems (Carpathians, Stara Planina, Balkans) (Stojanović and Karaman 2005, Stojanović and Karaman 2006, Csuzdi et al. 2006, Stojanović et al. 2008, Csuzdi et al. 2011, Pop et al. 2011, Carpenter et al. 2012, Melody and Schmidt 2012, Stojanović et al. 2012, Szederjesi and Csuzdi 2012a, Szederjesi and Csuzdi 2012b, Valchovski 2012, Keith and Schmidt 2013, Hackenberger-Kutuzović and Hackenberger-Kutuzović 2013, Milutinović et al. 2013, Stojanović et al. 2013, Szederjesi 2013b, Szederjesi 2013a, Lehmitz et al. 2014, Stojanović et al. 2014, Szederjesi 2014, Szederjesi et al. 2014a, Szederjesi et al. 2014b, Szederjesi and Csuzdi 2015, Szederjesi 2015). The many new records, plus corrections of some unintentional omissions in the 2004 release (e.g. *Dendrobaena cognettii* (Michaelsen, 1903) in Great Britain; Sims and Gerard 1985 as *D. pygmaea*), fall into the following geographical units: AL, BA, BG, DE, GB-GRB, GR-GRC, HR, HU, IE, MK, PT-POR, RO, YU.

**Family Megascolecidae**

On European coverage: *Pithemera bicincta* new records: greenhouses in Hungary (Csuzdi et al. 2008) and England (Sherlock and Carpenter 2009). *Metapheretima taprobanae* (Beddard, 1892) *first European record* in greenhouses at Kew, England (Sherlock and Carpenter 2009). *Pontodrilus litoralis* first report for Greece, from a rocky seashore in Thrace (Szederjesi 2015).

**Family Ocnerodrilidae**

On European coverage: *Eukerria saltensis* new records: France (Corsica) FR-COR, England GB-GRB (Rota 2013a). *Ocnerodrilus occidentalis* new records: Italian mainland IT-ITA (Rota 2013a), Greece (Szederjesi 2015), Jordan (Csuzdi and Pavlíček 2005) but already given as cosmopolitan.

**Family Octochaetidae**

On European coverage: *Dichogaster bolaui* new records: Ireland (Rota and Schmidt 2006), Hungary, Israel (Csuzdi et al. 2008).

**Family Propappidae**

On global coverage: *Propappus volki* new record: Japan (Torii 2006). This species has also been recorded in the St. Lawrence River in Canada (K.A. Coates, in Torii 2006).

**Family Sparganophilidae**

On European coverage: *Sparganophilus tamesis* new records: Germany (Graefe and Beylich 2011), Italy (Rota et al. 2014a), Switzerland (as *Sparganophilus langi* Bouché and Qiu 1998).

### Coordinates

Mediterranean (N 35°) and Arctic Islands (N 82°) Latitude; Atlantic Ocean (Mid-Atlantic Ridge) (W 30°) and Ural (E 60°) Longitude.

## Taxonomic coverage

### Description

The Fauna Europaea database contains the scientific names of all living European land and freshwater animal species, including numerous infra-groups and synonyms. More details about the conceptual background of Fauna Europaea and standards followed are described above and in the project description paper(s).

**Figure 6.**
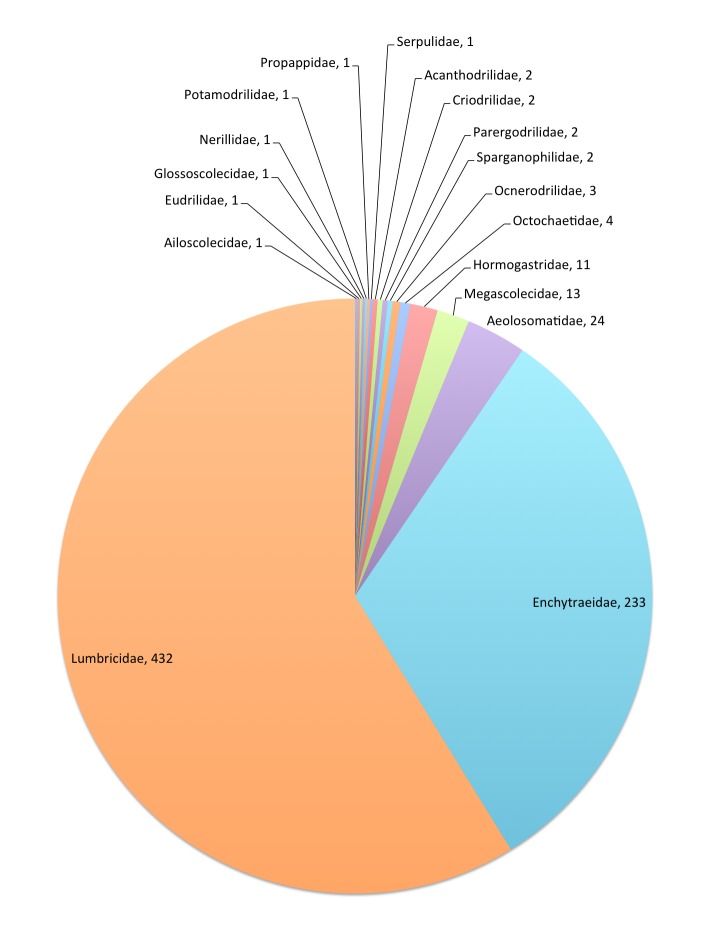
FaEu Annelida-Oligochaeta (terrestrial: Enchytraedae and Megadrili), Polychaeta and Aphanoneura species per family (see Table 1).

This data paper covers the Annelida Oligochaeta (terrestrial: Enchytraeidae and Megadrili), Aphanoneura and Polychaeta content of Fauna Europaea, including 18 families, updated from 735 species and 43 (sub)species synonyms (see Fig. 6, Table 1) to about 800 species.

Additional notes and details of updating information on the taxonomic status and coverage of Annelida – Oligochaeta (terrestrial), Aphanoneura and Polychaeta in Fauna Europaea can be found below:

**Family Aeolosomatidae**

According to Timm (2009), *Aeolosoma gineti* Juget, 1959 is not a member of the family, but possibly an oligochaete parvidrilid. Martinez-Ansemil et al. (2012) have removed it from Aeolosomatidae and placed it in Parvidrilidae (*Parvidrilus gineti*) as *species inquirenda*.

The correct year of publication of *A. tenebrarum* by Vejdovský is 1882.

**Family Enchytraeidae**

**1. Taxonomic novelties**

A useful key to the European terrestrial enchytraeids has recently been published by Schmelz and Collado (2010), Schmelz and Collado (2012). Some nomenclature changes proposed therein, however, are not adopted here because they appear unjustified, e.g. the relegation of some species to junior synonyms or nomina dubia (see Rota 2015), or the lumping of distinct lineages into imprecisely diagnosed taxa (*Cognettia sphagnetorum*; see Martinsson et al. 2014).

The taxonomic novelties considered here are the following:

**1.1. Newly described genera**

*Globulidrilus* Christensen & Dózsa-Farkas, 2012

**1.2. Newly described species**

*Achaeta antefolliculata* Dózsa-Farkas & Boros, 2005

*Achaeta borbonica* Rota, 2015

*Achaeta coimbrensis* Schmelz & Collado, 2013

*Achaeta diddeni* Graefe, 2007

*Achaeta giustii* Rota, 2015

*Achaeta unibulba* Graefe, Dózsa-Farkas & Christensen, 2009

*Cernosvitoviella longiducta* Dumnicka, 2010

*Cernosvitoviella tridentina* Dumnicka, 2004

*Chamaedrilus chalupskyi* Martinsson, Rota & Erséus, 2014

*Chamaedrilus pseudosphagnetorum* Martinsson, Rota & Erséus, 2014

*Chamaedrilus varisetosus* Martinsson, Rota & Erséus, 2015

*Cognettia valeriae* Dumnicka, 2010

*Fridericia argillae* Schmelz, 2003

*Fridericia bargaglii* Rota, 2015

*Fridericia brunensis* Schlaghamerský, 2007

*Fridericia ciliotheca* Schmelz & Collado, 2013

*Fridericia crassiductata* Dózsa-Farkas & Cech, 2006

*Fridericia cusanica* Schmelz, 2003

*Fridericia dozsae* Schmelz, 2003

*Fridericia granosa* Schmelz, 2003

*Fridericia gyromonodactyla* Boros & Dózsa-Farkas, 2015

*Fridericia healyae* Schmelz, 2003 (pro *F. polychaeta* Bretscher, 1900 sensu Southern 1907, Healy 1979)

*Fridericia lacii* Dózsa-Farkas, 2009

*Fridericia larix* Schmelz & Collado, 2005

*Fridericia longeaurita* Boros & Dózsa-Farkas, 2015

*Fridericia lenta* Schmelz, 2003 (pro *F. leydigii* (Vejdovský, 1878) sensu Nielsen and Christensen 1959)

*Fridericia mahunkai* Dózsa-Farkas, 2013

*Fridericia marginata* Schmelz & Collado, 2013

*Fridericia meridiana* Rota, 2015

*Fridericia rara* Rota, 2015

*Fridericia roembkei* Schmelz & Collado, 2013

*Fridericia schmelzi* Cech & Dózsa-Farkas, 2005

*Fridericia sousai* Schmelz & Collado, 2013

*Fridericia transylvanica* Boros & Dózsa-Farkas, 2015

*Marionina deminuta* Rota, 2013

*Marionina mendax* Rota, 2013

*Marionina mimula* Rota, 2013

*Marionina scintillans* Boros & Dózsa-Farkas, 2008

*Marionina sexdiverticulata* Dózsa-Farkas, 2002

**1.3. New names and synonymies**

*Bryodrilus librus* (Nielsen & Christensen, 1959) <= *B. parvus* Nurminen, 1970 jun. syn.

*Cernosvitoviella aggtelekiensis* Dózsa-Farkas, 1970 <= *C. goodhui* Healy, 1975 jun. syn.

*Cernosvitoviella palustris* Healy, 1979 <= *C. estaragniensis* Giani, 1979 jun. syn.

*Enchytraeus dichaetus* Schmelz & Collado, 2010 nom. nov. pro *E. minutus bisetosus* Rota & Healy, 1994

*Fridericia minor* Friend, 1913 <= *F. gracilis* von Bulow, 1957 jun. syn.

*Fridericia miraflores* Sesma & Dózsa-Farkas, 1996 <= *F. sylvatica* Healy, 1979 nom. dub.

*Mesenchytraeus pelicensis* Issel, 1905 <= *M. kuehnelti* Dózsa-Farkas, 1991 jun. syn.

*Fridericia* Michaelsen, 1889 <= *Timmodrilus* Dózsa-Farkas, 1997 jun. syn.

**1.4. New combinations**

*Bryodrilus librus* (Nielsen & Christensen, 1959)

*Chamaedrilus anomalus* (Černosvitov, 1928)

*Chamaedrilus cognettii* (Issel, 1905)

*Chamaedrilus glandulosus* (Michaelsen, 1888)

*Chamaedrilus hibernicus* (Healy, 1975)

*Chamaedrilus lapponicus* (Nurminen, 1965)

*Chamaedrilus paxi* (Moszyński, 1938)

*Chamaedrilus sphagnetorum* (Vejdovský, 1878)

*Chamaedrilus valeriae* (Dumnicka, 2010)

*Euenchytraeus clarae* (Bauer, 1993)

*Globulidrilus riparius* (Bretscher, 1899)

**1.5. Reinstatements as valid names**

*Chamaedrilus* Friend, 1913 pro *Cognettia* Nielsen & Christensen, 1959 (partim)

*Euenchytraeus* Bretscher, 1906 pro *Cognettia* Nielsen & Christensen, 1959 (partim)

*Buchholzia subterranea* (Černosvitov, 1937)

*Chamaedrilus chlorophilus* Friend, 1913

*Enchytraeus bohemicus* Dumnicka, 1996

*Fridericia digitata* Cognetti, 1901

*Fridericia glandifera* Friend, 1913

*Fridericia humicola* Bretscher, 1900

**1.6. Species complexes**

The following names are considered as species complexes, their taxonomy being currently still unresolved:

*Enchytraeus buchholzi* s.l.

*Enchytronia parva* s.l.

*Fridericia aurita* s.l.

*Fridericia ratzeli* s.l.

**1.7. Rejected synonymies**

*Achaeta etrusca* Rota, 1995 ≠ *A. iberica* Graefe, 1989

*Fridericia caprensis* Bell, 1947 ≠ *F. pretoriana* Stephenson, 1930

*Fridericia sohlenii* Rota, Healy & Erséus, 1998 ≠ *F. cylindrica* Springett, 1971

*Fridericia glandulosa* Southern, 1907 ≠ *F. galba* (Hoffmeister, 1843)

**Family Hormogastridae**

The correct year of publication of *Vignysa* Bouché is 1970; same for *Vignysa popi* Bouché, 1970 (both recorded as 1972 in the 2004 release).

Taxonomic comments: *H. elisae* species complex comprises at least five cryptic allopatric species.

Species to be added as valid: *H. riojana*, *H. ireguana*, *H. eserana*, *H. huescana*, *H. arenicola*, *H. catalaunensis*, *H. sylvestris*, *H. najaformis*, *H. castillana* (no *H. multilamella*, *H. lleidana*), all authored 'Qiu & Bouché, 1998'

*Hormogaster abbatissae* Novo et al., 2012a

*Hormogaster joseantonioi* Fernández Marchán et al., 2014

**Family Lumbricidae**

**1. Taxonomic novelties**

Since the first version of the Fauna Europaea database in 2004, some 17 new species have been described from our continent and the status of some known species has been formally revised (Perez-Onteniente and Rodriguez-Babio 2004, Zicsi and Cuendet 2005, Csuzdi and Pop 2008, Höser and Zicsi 2009, Perez-Onteniente and Rodriguez-Babio 2010, Csuzdi et al. 2011, Blakemore 2012a, Szederjesi and Csuzdi 2012a, Szederjesi and Csuzdi 2012b, Díaz-Cosín et al. 2014, Szederjesi et al. 2014a, Szederjesi and Csuzdi 2015). Considering the uncertain phylogenetic status of many genera, recently published proposals of new genera and genus rank (e.g. Blakemore 2012a for *Prosellodrilus* subgenera) will be not considered in the present FaEu database updating.

**1.1. Newly described species**:

*Allolobophora prosellodacica* Csuzdi & Pop, 2008 (herein transferred to *Eophila*)

*Allolobophora ruzsai* Szederjesi, 2014

*Dendrobaena luraensis* Szederjesi & Csuzdi, 2012

*Dendrobaena retrosella* Szederjesi & Csuzdi, 2012

*Dendrobaena virgata* Szederjesi et al., 2014

*Dendrobaena vladeasa* Csuzdi et al., 2011

*Eisenia muranyii* Szederjesi & Csuzdi, 2015

*Eisenia oreophila* Szederjesi & Csuzdi, 2012

*Eiseniona gerardoi* Díaz-Cosín et al., 2014

*Eumenescolex proclitellatus* Perez-Onteniente & Rodriguez-Babio, 2004

*Kenleenus armadas* Blakemore, 2012 (pro *Prosellodrilus amplisetosus* Bouché, 1972 sensu Melody and Schmidt 2012, Keith and Schmidt 2013) (herein transferred to *Prosellodrilus*)

*Octodrilus albanicus* Szederjesi & Csuzdi, 2015

*Octodrilus izanus* Csuzdi et al., 2011

*Octodrilus juvyi* Zicsi & Cuendet, 2005

*Octodrilus parvivesiculatus* Csuzdi et al., 2011

*Proctodrilus thaleri* Höser & Zicsi, 2009

*Zophoscolex albacetensis* Perez-Onteniente & Rodriguez-Babio, 2010 (herein transferred to *Aporrectodea*)

**1.2. New synonymies**:

*Eophila getica* (Pop, 1947) <= Allolobophora dugesi var. getica = Cernosvitovia (Zicsiona) getica Mršić & Šapkarev, 1987 = Cernosvitovia (Zicsiona) silicata Mršić & Šapkarev, 1987 = Cernosvitovia (Zicsiona) paradoxa Mršić, 1992; revision by Csuzdi and Pop (2007b).

*Dendrobaena attemsi* Michaelsen, 1902 <= *Dendrobaena apora* Qiu & Bouché, 1998; revision by Szederjesi and Csuzdi (2012a).

*Octolasion cyaneum* (Savigny, 1826) <= *Dendrobaena jeanneli* Pop, 1948 revision by Csuzdi and Pop (2007a).

**1.3. New combinations**

*Aporrectodea albacetensis* (Perez-Onteniente & Rodriguez-Babio, 2010)

*Eophila prosellodacica* (Csuzdi & Pop, 2008)

*Prosellodrilus armadas* (Blakemore, 2012)

**Family Sparganophilidae**

*Sparganophilus langi* Qiu & Bouché, 1998 from Switzerland is a junior synonym of *S. tamesis* Benham, 1892 (Graefe and Beylich 2011, Rota et al. 2014a).

**2. Classification**

The oligochaete suprafamilial rankings, as they were published in FaEu 2004 and listed below, need revision. No adjustments to the rankings were introduced in this paper.

### Taxa included

**Table:** 

Rank	Scientific Name	Common Name
kingdom	Animalia	
subkingdom	Eumetazoa	
phylum	Annelida	
class	Aphanoneura	
family	Aeolosomatidae	
family	Potamodrilidae	
class	Oligochaeta	
subclass	Diplotesticulata	
superorder	Megadrili	
order	Opisthopora	
suborder	Lumbricina	
superfamily	Criodriloidea	
family	Criodrilidae	
superfamily	Eudriloidea	
family	Eudrilidae	
superfamily	Lumbricoidea	
family	Ailoscolecidae	
family	Glossoscolecidae	
family	Hormogastridae	
subfamily	Hormogastrinae	
subfamily	Vignysinae	
subfamily	Xaninae	
family	Lumbricidae	
subfamily	Diporodrilinae	
subfamily	Lumbricinae	
subfamily	Spermophorodrilinae	
superfamily	Megascolecoidea	
family	Acanthodrilidae	
family	Megascolecidae	
family	Ocnerodrilidae	
family	Octochaetidae	
superfamily	Sparganophiloidea	
family	Sparganophilidae	
subclass	Tubificata	
order	Tubificida	
suborder	Enchytraeina	
superfamily	Enchytraeoidea	
family	Enchytraeidae	
family	Propappidae	
class	Polychaeta	
order	Nerillida	
family	Nerillidae	
order	Sabellida	
family	Serpulidae	
subclass	Polychaeta incertae sedis	
family	Parergodrilidae	

## Temporal coverage

**Living time period:** Currently living.

### Notes

Currently living animals in stable populations, largely excluding (1) rare/irregular immigrants, intruder or invader species, (2) accidental or deliberate releases of exotic (pet) species, (3) domesticated animals, (4) foreign species imported and released for bio-control or (5) foreign species largely confined to hothouses.

## Usage rights

### Use license

Open Data Commons Attribution License

### IP rights notes

Fauna Europaea data are licensed under CC BY SA version 4.0. The property rights of experts over their data is covered by their Fauna Europaea contract agreements. For more IPR details see: http://www.faunaeur.org/copyright.php.

## Data resources

### Data package title

Fauna Europaea - Annelida-Oligochaeta

### Resource link

http://www.faunaeur.org/Data_papers/FaEu_Annelida-Oligochaeta_2.6.2.zip

### Alternative identifiers

http://www.faunaeur.org/experts.php?id=101

### Number of data sets

2

### Data set 1.

#### Data set name

Fauna Europaea - Annelida-Oligochaeta version 2.6.2 - species

#### Data format

CSV

#### Number of columns

25

#### Character set

UTF-8

#### Download URL

http://www.faunaeur.org/Data_papers/FaEu_Annelida-Oligochaeta_2.6.2.zip

#### Description

**Data set 1.** 

Column label	Column description
datasetName	The name identifying the data set from which the record was derived (http://rs.tdwg.org/dwc/terms/datasetName).
version	Release version of data set.
versionIssued	Issue data of data set version.
rights	Information about rights held in and over the resource (http://purl.org/dc/terms/rights).
rightsHolder	A person or organization owning or managing rights over the resource (http://purl.org/dc/terms/rightsHolder).
accessRights	Information about who can access the resource or an indication of its security status (http://purl.org/dc/terms/accessRights).
taxonID	An identifier for the set of taxon information (http://rs.tdwg.org/dwc/terms/taxonID)
parentNameUsageID	An identifier for the name usage of the direct parent taxon (in a classification) of the most specific element of the scientificName (http://rs.tdwg.org/dwc/terms/parentNameUsageID).
scientificName	The full scientific name, with authorship and date information if known (http://rs.tdwg.org/dwc/terms/scientificName).
acceptedNameUsage	The full name, with authorship and date information if known, of the currently valid (zoological) taxon (http://rs.tdwg.org/dwc/terms/acceptedNameUsage).
originalNameUsage	The original combination (genus and species group names), as firstly established under the rules of the associated nomenclaturalCode (http://rs.tdwg.org/dwc/terms/originalNameUsage).
family	The full scientific name of the family in which the taxon is classified (http://rs.tdwg.org/dwc/terms/family).
familyNameId	An identifier for the family name.
genus	The full scientific name of the genus in which the taxon is classified (http://rs.tdwg.org/dwc/terms/genus).
subgenus	The full scientific name of the subgenus in which the taxon is classified. Values include the genus to avoid homonym confusion (http://rs.tdwg.org/dwc/terms/subgenus).
specificEpithet	The name of the first or species epithet of the scientificName (http://rs.tdwg.org/dwc/terms/specificEpithet).
infraspecificEpithet	The name of the lowest or terminal infraspecific epithet of the scientificName, excluding any rank designation (http://rs.tdwg.org/dwc/terms/infraspecificEpithet).
taxonRank	The taxonomic rank of the most specific name in the scientificName (http://rs.tdwg.org/dwc/terms/infraspecificEpithet).
scientificNameAuthorship	The authorship information for the scientificName formatted according to the conventions of the applicable nomenclaturalCode (http://rs.tdwg.org/dwc/terms/scientificNameAuthorship).
authorName	The four-digit year in which the scientificName was published (http://rs.tdwg.org/dwc/terms/namePublishedInYear).
namePublishedInYear	The four-digit year in which the scientificName was published (http://rs.tdwg.org/dwc/terms/namePublishedInYear).
Brackets	Annotation if authorship should be put between parentheses.
nomenclaturalCode	The nomenclatural code under which the scientificName is constructed (http://rs.tdwg.org/dwc/terms/nomenclaturalCode).
taxonomicStatus	The status of the use of the scientificName as a label for a taxon (http://rs.tdwg.org/dwc/terms/taxonomicStatus).
resourceDescription	An account of the resource, including a data-paper DOI (http://purl.org/dc/terms/description)

### Data set 2.

#### Data set name

Fauna Europaea - Annelida-Oligochaeta version 2.6.2 - hierarchy

#### Data format

CSV

#### Number of columns

12

#### Character set

UTF-8

#### Download URL

http://www.faunaeur.org/Data_papers/FaEu_Annelida-Oligochaeta_2.6.2.zip

#### Description

**Data set 2.** 

Column label	Column description
datasetName	The name identifying the data set from which the record was derived (http://rs.tdwg.org/dwc/terms/datasetName).
version	Release version of data set.
versionIssued	Issue data of data set version.
rights	Information about rights held in and over the resource (http://purl.org/dc/terms/rights).
rightsHolder	A person or organization owning or managing rights over the resource (http://purl.org/dc/terms/rightsHolder).
accessRights	Information about who can access the resource or an indication of its security status (http://purl.org/dc/terms/accessRights).
taxonName	The full scientific name of the higher-level taxon
scientificNameAuthorship	The authorship information for the scientificName formatted according to the conventions of the applicable nomenclaturalCode (http://rs.tdwg.org/dwc/terms/scientificNameAuthorship).
taxonRank	The taxonomic rank of the most specific name in the scientificName (http://rs.tdwg.org/dwc/terms/infraspecificEpithet).
taxonID	An identifier for the set of taxon information (http://rs.tdwg.org/dwc/terms/taxonID)
parentNameUsageID	An identifier for the name usage of the direct parent taxon (in a classification) of the most specific element of the scientificName (http://rs.tdwg.org/dwc/terms/parentNameUsageID).
resourceDescription	An account of the resource, including a data-paper DOI (http://purl.org/dc/terms/description)

## Figures and Tables

**Table 1.** Responsible specialists per family in Annelida Oligochaeta (terrestrial: Enchytraeidae and Megadrili), Aphanoneura and Polychaeta. The numbers of databased species is given per family (see also Fig. 6) plus the actual number of known/described species (showing a potential information gap).

TAXONOMY			EUROPE		
CLASS	FAMILY	SPECIALIST(S)	DATABASED SPECIES (Fauna Europaea)	TOTAL DESCRIBED SPECIES (information-gap)	COMMENT
Aphanoneura	Aeolosomatidae	Emilia Rota	24	23	*Aeolosoma gineti* transferred to the oligochaete family Parvidrilidae
Aphanoneura	Potamodrilidae	Emilia Rota	1	1	
Polychaeta	Nerillidae	Emilia Rota	1	1	
Polychaeta	Parergodrilidae	Emilia Rota	2	2	Including *Hrabeiella periglandulata*, incertae sedis species
Polychaeta	Serpulidae	Emilia Rota	1	1	
Oligochaeta	Acanthodrilidae	Emilia Rota	2	2	
Oligochaeta	Ailoscolecidae	Emilia Rota	1	1	
Oligochaeta	Criodrilidae	Emilia Rota	2	2	
Oligochaeta	Enchytraeidae	Emilia Rota	233	274	By addition of 2 new records, 39 new species, 6 synonymies, 6 reinstated species
Oligochaeta	Eudrilidae	Emilia Rota	1	1	
Oligochaeta	Glossoscolecidae	Emilia Rota	1	1	
Oligochaeta	Hormogastridae	Emilia Rota	11	22	By addition of 2 new species and 9 reinstated species
Oligochaeta	Lumbricidae	Emilia Rota	432	446	By addition of 17 new species and 3 synonymies
Oligochaeta	Megascolecidae	Emilia Rota	13	14	One new record in greenhouses
Oligochaeta	Ocnerodrilidae	Emilia Rota	3	3	
Oligochaeta	Octochaetidae	Emilia Rota	4	4	
Oligochaeta	Propappidae	Emilia Rota	1	1	
Oligochaeta	Sparganophilidae	Emilia Rota	2	1	By deletion of 1 synonym
